# Genetic Parameter Estimation and Genome-Wide Association Analysis of Social Genetic Effects on Average Daily Gain in Purebreds and Crossbreds

**DOI:** 10.3390/ani12172300

**Published:** 2022-09-05

**Authors:** Ha-Seung Seong, Young-Sin Kim, Soo-Jin Sa, Yongdae Jeong, Joon-Ki Hong, Eun-Seok Cho

**Affiliations:** National Institute of Animal Science, Rural Development Administration, Cheonan 31000, Korea

**Keywords:** social genetic effect, average daily gain, purebred, crossbred

## Abstract

**Simple Summary:**

Average daily gain (ADG) is influenced by both an individual’s direct genetic effect (DGE) and by a social genetic effect (SGE) derived from pen mates. Therefore, identifying the DGE and SGE on ADG is essential for a better understanding of pig breeding systems. We conducted this study to elucidate the genetic characteristics and relationships of DGE and SGE on ADG using purebred and crossbred pigs. We found that the DGE and SGE both contributed to ADG in both populations. In addition, the SGE of purebred pigs was highly correlated with the DGE of crossbred pigs. Furthermore, we identified several genomic regions that may be associated with the DGE and SGE on ADG. Our findings will contribute to future genomic evaluation studies of socially affected traits.

**Abstract:**

Average daily gain (ADG) is an important growth trait in the pig industry. The direct genetic effect (DGE) has been studied mainly to assess the association between genetic information and economic traits. The social genetic effect (SGE) has been shown to affect ADG simultaneously with the DGE because of group housing systems. We conducted this study to elucidate the genetic characteristics and relationships of the DGE and SGE of purebred Korean Duroc and crossbred pigs by single-step genomic best linear unbiased prediction and a genome-wide association study. We used the genotype, phenotype, and pedigree data of 1779, 6022, and 7904 animals, respectively. Total heritabilities on ADG were 0.19 ± 0.04 and 0.39 ± 0.08 for purebred and crossbred pigs, respectively. The genetic correlation was the greatest (0.77 ± 0.12) between the SGE of purebred and DGE of crossbred pigs. We found candidate genes located in the quantitative trait loci (QTLs) for the SGE that were associated with behavior and neurodegenerative diseases, and candidate genes in the QTLs for DGE that were related to body mass, size of muscle fiber, and muscle hypertrophy. These results suggest that the genomic selection of purebred animals could be applied for crossbred performance.

## 1. Introduction

In the swine production industry, many pigs can be managed in a house grouping system. The development of piglets is known to be substantially affected by group mates in such a system [1]. Socially and physically enriched pens have been shown to have beneficial effects on productive traits, such as increased feed intake and weight, compared with pens that are not enriched [2]. However, group mates can have disadvantageous effects on production, and the introduction of new pigs into previously existing groups usually results in physically damaging events [3]. Therefore, social interaction within groups is considered as one of the factors that can affect productive traits. The concept of the indirect genetic effect was first introduced by Griffing [4] to describe the effect of an individual’s genotype on the traits of others in the same group; this is also called the social genetic effect (SGE) [5]. Another genetic effect is direct genetic effect (DGE), which describes the effect of an individual’s genotype on its phenotype [3].

Average daily gain (ADG) is an important indicator of growth performance in pigs, and many genome-wide association studies (GWAS) have been conducted to better understand the relationship between genetic information and ADG [6,7,8]. The association of the SGE with ADG has been widely studied using diverse analysis approaches [5,9,10,11]. One of the most popular methods for genomic prediction is single-step best linear unbiased prediction (ssGBLUP), which enables the use of phenotype information of both individuals with and without genotypes for genomic prediction. ssGBLUP uses a hybrid matrix (H) that is generated by combining a relationship matrix based on pedigree (A) with the genomic relationship (G) [12,13]. Subsequently, a weighted ssGBLUP method, based on the application of different single-nucleotide polymorphism (SNP) weights, was proposed [14].

In the livestock industry, genomic selection for productive traits is used to rapidly improve livestock. Genomic prediction studies for the ADG trait have been extensively conducted in different pig populations [11,15,16,17,18]. Genomic selection events in purebred pigs have been suggested to increase the selection response for performance in crossbred pigs [19], and Ask et al. [11] have recently shown that selection for the SGE on ADG in purebred pigs can improve ADG in two-way crossbred pigs. However, there is still a lack of studies that elucidate the genetic relationship of the DGE and SGE between purebred and crossbred pigs. In this study, we used purebred Korean Duroc and crossbred pigs that are crossed using Korean Duroc and Korean native pigs to take advantage of both populations regarding their growth rate and meat quality. The purposes of this study are to (1) estimate the variance components for the DGE and SGE in purebred and crossbred populations; (2) estimate the genetic correlation between the SGE or DGE on ADG in purebred and crossbred pigs; and (3) perform GWAS to identify candidate genes associated with the DGE and SGE on ADG in pigs.

## 2. Materials and Methods

### 2.1. Animals, Pedigree and Phenotype Data

The pedigree data of 7904 animals, including 5408 purebred Korean Duroc (DUC) [20], 21 Korean native pigs (KNP), and 2475 crossbred pigs, were collected from 2001 to 2020. The crossbred pigs (F1 × F2) were generated by the breeding scheme described previously [20,21]; using DUC and KNP as the parental breeds, and F1 (DUC × KNP) and F2 (F1 × DUC). The theoretical genetic composition of the crossbred animals is 62.5% and 37.5% from DUC and KNP, respectively [21]. The phenotype data were obtained from 6022 animals, including 3858 DUC and 2164 crossbred pigs.

ADG (g/day) was measured from 30 kg (start weight) to 100 kg (end weight) as follows.

### 2.2. Genotype Data

Genomic DNA was extracted from blood or hair root samples and genotyped using an Illumina porcineSNP60K BeadChip v2 (Illumina, Inc., San Diego, CA, USA), which includes 61,565 SNPs for 1779 animals (864 DUC and 915 crossbreds). The quality control process was conducted for SNP markers and animals as the following criteria: (1) SNPs unmapped in *Sus scrofa* 11.1 or sex chromosomes; (2) SNPs with a call rate < 90%; (3) SNPs with minor allele frequency < 0.05; (4) monomorphic SNPs; (5) animals with a call rate < 90%; (6) animals with Mendelian conflicts. This quality control process removed 18,871 SNPs and 12 animals, leaving a total of 42,694 SNPs for 1767 animals for further analyses.

### 2.3. Genetic Parameters and Variance Components

Variance and covariance components for the ADG trait for the DUC and crossbred pigs were estimated using the multi-trait model based on the Bayesian approach in GIBBS2F90 [22]. The Gibbs sampler was run a total of 120,000 rounds with single chains, and the first 20,000 rounds were excluded as burn-in rounds, thinning every 10 samples. Consequently, we used 10,000 samples for the subsequent post-Gibbs analysis in POSTGIBBSF90 [22].

Sex (male or female), birth year–2 months (54 levels), and group size (6 levels) were used as the fixed effects, and start weight (g) and age at target weight (100 kg) were fitted as covariates. Group (2246 levels), birth litter (1478 levels), and animal (7904 levels) were used as random effects. Genetic analysis was performed using the animal model as follows:

$$y=Xb+Z_{D}a_{D}+Z_{S}a_{S}+Td+Ul+e$$

where y is the vector of ADG, *b* is the vector of fixed effects, $a_{D}$ and $a_{S}$ are the vectors of the random additive DGE and SGE, respectively, *d* is the vector of the random group with $d\;\sim \;N\left(0,I\sigma_{d}^{2}\right)$, *l* is the vector of random birth litter, *e* is the vector for the residuals, $e\;\sim \;N\left(0,I\sigma_{e}^{2}\right)$, and *X,* $Z_{D}$*,* $Z_{S}$*, T,* and *U* are the corresponding incidence matrixes. Because the pen sizes were different, we added a dilution factor (average group size−1)/(group size−1) to the SGE. The genetic correlations for pairwise genetic effects (DGE and SGE) on ADG in both DUC and crossbred were also estimated.

For the ADG trait affected by both heritable DGE and SGE, the variances in total breeding value (TBV) were estimated as follows [23]:

$$\sigma_{TBV}^{2}=\sigma_{a_{D}}^{2}+2\left(n-1\right)\sigma_{a_{D}a_{S}}+{\left(n-1\right)}^{2}\sigma_{a_{S}}^{2}$$

In addition, the TBV for the *i*-th individual was calculated as described by Bijma [23], using the following equation:

$$TBV_{i}=a_{D,i}+\left(n-1\right)a_{S,i}$$

where *n* is the average pen size, $a_{D,i}$ and $a_{S,i}$ are the sum of DGE and SGE, respectively.

The phenotypic variance for the multi-trait model was obtained as follows:

$$\sigma_{p}^{2}=\sigma_{a_{D}}^{2}+\left(n-1\right)\sigma_{a_{S}}^{2}+\sigma_{d}^{2}+\sigma_{l}^{2}+\sigma_{e}^{2}$$

Total heritability ($T^{2}$) was estimated as follows:

$$T^{2}=\frac{\sigma_{TBV}^{2}}{\sigma_{p}^{2}}$$

### 2.4. Single-Step Genome-Wide Association Study

We performed a GWAS on random additive DGE and SGE [5] using the ssGBLUP approach [12,24], which considered all the phenotype, genotype, and pedigree data in a single step. The ssGBLUP uses a realized relationship matrix (*H* matrix) that combines genomic and pedigree information. The relationship among the matrices is as follows:

$$H^{-1}=A^{-1}+\left[\begin{array}{cc} 0 & 0\\ 0 & G^{-1}-A_{22}^{-1} \end{array}\right]$$

where $A^{-1}$ is the inverse of the numerator relationship matrix, $G^{-1}\;$is the inverse of the genomic relationship matrix, and $A_{22}^{-1}$ is the inverse of the pedigree relationship matrix. We obtained the *G* weight matrix generated by reciprocals of expected variance of markers as proposed by VanRaden [25] as follows:

(1) $$G=ZDZ^{\prime }q$$

where *Z* is the incidence matrix of genetic content that is altered for allele frequencies, *D* is the diagonal weight matrix of SNPs, and *q* is a normalizing factor. The effects and weights of the SNPs were obtained as follows:

1. D = I in the first step;
2. Calculation of breeding values;
3. Calculation of SNP effects, $\hat{u}=DZ^{\prime }{\left[ZDZ^{\prime }\right]}^{-1}{\hat{a}}_{g}$, where ${\hat{a}}_{g}$ is the breeding value for genotyped individuals;
4. Calculation of SNP weight for each SNP, $d_{i}={\hat{u}}^{2}2p_{i}\left(1-p_{i}\right)$, where *i* is the *i*-th SNP;
5. Normalization of SNP weight for retaining constant total genetic variance;
6. Then loop to step 2.

This process was run for three iterations and SNP effects, breeding values, and the *D* matrix were recalculated as described by Wang et al. [14]. In this study, we grouped SNPs located within 0.4 Mb as a single window, and the percentage of genetic variance explained by each window was calculated using the postGSF90 module for association analysis as follows [26]:

(2) $$\frac{Var\left(a_{i}\right)}{\sigma_{a}^{2}}\times 100=\frac{Var\left(\sum_{j=1}^{x}Z_{j}{\hat{u}}_{j}\right)}{\sigma_{a}^{2}}\times 100$$

where $a_{i}$ was the genetic value of the *i*-th region consisting of *x* = 0.4 Mb.

### 2.5. Candidate Genes and Gene Ontology (GO)

To identify the candidate genes associated with DGE and SGE in the crossbred and purebred pig populations, we first determined the threshold for significant SNPs that explained >0.4% of the additive genetic variance. Then, 1-Mb regions that had significant SNPs in their centers were defined as quantitative trait loci (QTLs). We annotated genes within the QTLs based on the *Sus scrofa* genome assembly 11.1 (https://ftp.ncbi.nlm.nih.gov/genomes/all/GCF/000/003/025/GCF_000003025.6_Sscrofa11.1/GCF_000003025.6_Sscrofa11.1_genomic.gff.gz, accessed on 16 March 2022). We also mapped the QTLs discovered in this study to previously reported pig QTLs to identify the overlapping regions using the Pig QTL Database (https://www.animalgenome.org/cgi-bin/QTLdb/SS/index, accessed on 2 June 2022). Gene Ontology (GO) and Kyoto Encyclopedia of Genes and Genomes (KEGG) pathway analyses were performed using ClueGO v2.5.9 and CluePedia v1.5.9 plug-ins in Cytoscape (v3.9.1) [27,28]. GO terms, with a Bonferroni step-down adjusted *p*-value ≤ 0.05, were considered to be significantly enriched and were used to annotate the candidate genes. The GeneCards database (https://www.genecards.org/, accessed on 9 June 2022) was used to retrieve phenotype information of the annotated genes.

## 3. Results and Discussion

### 3.1. ADG Performance, Genetic Parameters and Variance Components

The average ADG (g) was higher in the DUC (986.04 ± 125.25) than it was in the crossbred (849.43 ± 110.87) pigs (Table S1). Duroc pigs are known to have undergone intensive artificial selection over 100 years, and have superior carcass, growth, and feed conversion efficiency traits compared with those of other breeds [29]. KNPs, one of the parental breeds of the crossbred pigs, have undergone severe inbreeding events because of their low population size [21], and their growth performance is also lower than that of commercial breeds. Therefore, the average ADG in the crossbred pigs was lower than it was in the DUC pigs. To better understand the genetic information of the ADG in the purebred and crossbred pigs, we estimated the direct and social genetic variance ($\sigma_{aD}^{2}$ and $\sigma_{aS}^{2}$), phenotypic variance ($\sigma_{p}^{2}$), total heritable variance ($\sigma_{TBV}^{2}$), direct heritability ($h^{2}$), total heritability ($T^{2}$), and genetic correlation between the DGE and SGE ($r_{D-S}$). The variance components of these genetic parameters in DUC and crossbred pigs are given in Table 1.

The $h^{2}$ values for ADG were 0.16 ± 0.04 and 0.36 ± 0.05 for DUC and crossbred pigs, respectively, and the $T^{2}$ values, which include both DGE and SGE, were slightly higher in both populations, with 0.19 ± 0.04 and 0.39 ± 0.08 for DUC and crossbred, respectively (Table 1). However, the crossbred pigs showed little difference in heritability values ($h^{2}$ and $T^{2}$) due to the negative correlation (−0.15 ± 0.27) between the direct and social genetic effects.

This finding confirmed the contribution of SGE to total heritable variance. We found that the DUC had lower heritability for ADG than the crossbred pigs, and they also had lower heritability for ADG than other Duroc populations reported previously [30,31,32,33]. These results suggest that intensive selection events for growth traits have occurred in DUC populations. Indeed, DUC pigs have been intensively selected for growth traits after they were introduced into South Korea. A recent study reported substantial genetic improvement of ADG in DUC pigs, as the estimated breeding value for ADG has increased from −5.23 g to 45.16 g since 2000 [30]. Therefore, there may now be less chance for genetic improvement for ADG in the DUC population. The genetic correlation between SGE and DGE was neutral for DUC (0.03 ± 0.20) and weak for crossbred (−0.15 ± 0.27) (Table 1). This result is consistent with the work of Bergsma et al. [34], who found that the absence of conflict between an individual’s growth and a mate’s growth may be a consequence of neutral or weak social interactions.

### 3.2. Genetic Correlations between Purebred and Crossbred Pigs

To observe the relationship between genetic effects, we estimated the genetic correlations between the DGE and SGE for DUC and crossbred pigs (Table 2). The genetic correlation between DGE of DUC and that of crossbred pigs was favorable (0.48 ± 0.19) and statistically significant (Table 2).

This result is similar to that of a previous report of statistically significant and moderate genetic correlations between the DGE on ADG of a crossbred dam (F1, landrace × Yorkshire) and that of landrace (0.46 ± 0.18) or Yorkshire (0.41 ± 0.17) [11]. The genetic correlation between the SGE of DUC and that of crossbred pigs was negative (−0.27 ± 0.25) and not statistically significant. The genetic correlation between the DGE for DUC and SGE for crossbreds was negative (−0.53 ± 0.23) and statistically significant. The estimated genetic correlation between SGE for DUC and DGE for crossbreds was the highest among the pairwise correlations (0.77 ± 0.12) and statistically significant, indicating that the SGE for DUC was highly associated with crossbred performance. Previous studies have also indicated that genomic selection of purebred animals can increase the selection response for crossbred performance [19,35]. Ask et al. [11] reported a positive effect of SGE that was assessed using purebred data on the ADG traits in F1 crossbred pigs. As we found moderate (between DGE of DUC and DGE of crossbred) to high genetic correlations (between SGE of DUC and DGE of crossbred) between the DUC and crossbred pigs, we suggest that genomic selection of the purebred population, especially for social behavior, may have affected the selection response for ADG in the crossbred population.

### 3.3. QTLs for DGE and SGE

**Figure 1:**
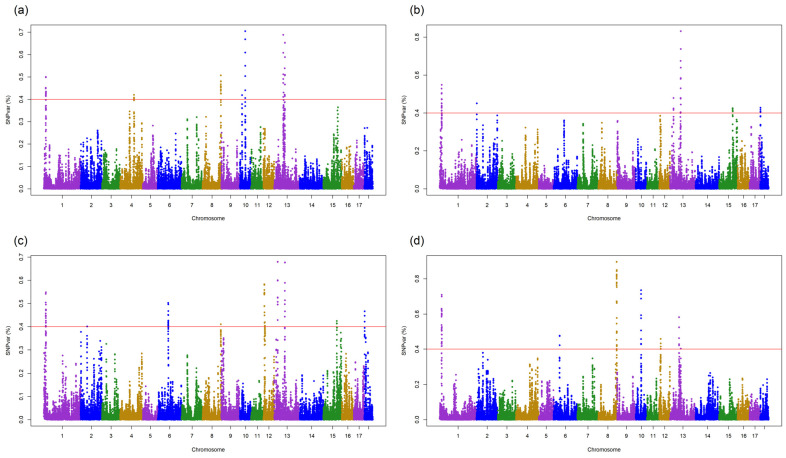
Results of single-step GWAS for DGE and SGE on average daily gain (ADG) in DUC and crossbred pigs. SNPvar (%) is the variance explained by SNPs within 0.4 Mb-sized windows: single-step GWAS plot for DGE on ADG in (**a**) DUC and (**b**) crossbred pigs; single-step GWAS plot for SGE on ADG in (**c**) DUC and (**d**) crossbred pigs. The horizontal line (red) represents the threshold of explained variance (0.4%).

As shown in Figure 1a and Table S2, the GWAS identified 52 significant SNPs (explained genetic variance > 0.4%) associated with DGE in DUC pigs.

These SNPs explained 24.9% of the total genetic variance and were located on SSC1, SSC4, SSC8, SSC10, and SSC13. The gene annotations for the 1-Mb QTLs that centered those SNPs identified 97 genes associated with these QTLs (Table S2). Because a large number of QTLs were detected in this study, we focused on the QTLs that explained the greatest genetic variance for the DGE and SGE in DUC and crossbred pigs and compared them with previously reported pig QTLs. A QTL in SSC10 (31.4–32.4 Mb) that explained the most genetic variance (0.7%) overlapped with the production QTLs that were previously reported to be associated with ADG [36,37], as well as with the QTLs associated with feed conversion ratio [38] and meat-related traits [39,40,41]. In the crossbred pigs, 43 significant SNPs (explained 20.5% of genetic variance) associated with DGE were located on SSC1, SSC2, SSC13, SSC15, and SSC18 (Figure 1b), and 83 genes were annotated within the corresponding QTLs (Table S2). We also found that the QTL (65.3–66.3 Mb) in SSC13 that explained the most genetic variance (0.8%) overlapped with the production-related QTLs that are associated with ADG [37], as well as meat-related QTLs that are associated with backfat weight, the percentage of loin fat, and loin muscle area [42].

For SGE in DUC, 70 significant SNPs located on SSC1, SSC2, SSC6, SSC8, SSC12, SSC13, SSC15, and SSC18 explained 33.6% of the total genetic variance (Figure 1c, Table S2). A QTL located on SSC13 (16.4–17.4 Mb) that explained the highest genetic variance (0.7%) overlapped with a QTL associated with the time spent socializing [43], as well as with production-related QTLs that are associated with ADG and chest width [36,37]. We also detected QTLs associated with meat-related traits, such as loin muscle area, average backfat thickness, and backfat at tenth rib [36,44]. For SGE in crossbred pigs, 76 significant SNPs located on SSC1, SSC6, SSC8, SSC10, SSC12, and SSC13 explained 42.8% of the total genetic variance (Figure 1d; Table S2). The top QTLs (SSC8, 136.3–137.3 Mb), which explained 0.9% of the genetic variance, overlapped with QTLs related to coping behavior [45] and exploration during stress [46], as well as with QTLs associated with the percentage of lean meat, rump width, and length of humerus [47,48].

### 3.4. GO and KEGG Analyses for SGE

SGE is an established concept in behavioral ecology [49]. SGE is not associated with one specific social interaction, but instead captures the overall effect of several social interactions between individuals on a specific trait of the recipient individual [50]. Hong et al. [5] identified positional candidate genes for SGE on ADG that have biological roles that are strongly associated with neuropsychiatric processes. We also identified candidate genes that may be related to the neurological disorders and behavioral changes.

We identified genes that were annotated with 17 GO terms that might be associated with the SGE in DUC (Table 3), including *DBX1*, *PAX7*, and *SHH*, which were annotated with the neuron fate commitment term (GO: 0048663). *DBX1* is expressed in hypothalamic progenitors and restriction of *DBX1* was found to be critical in establishing the neuronal fate of V0 and V1, which are derived from adjacent progenitor domains [51]. The previous study revealed that *DBX1* is associated with diminished responses to feeding stressors and abnormal GABAergic neuron morphology [51,52]. Proskorovski-Ohayon et al. [53] suggested that homozygous mutation in *PAX7* likely causes a neuromuscular syndrome in humans. Dysregulation of the SHH pathway in the brain was reported to be associated with neurodegenerative diseases, such as amyotrophic lateral sclerosis and Parkinson’s disease [54].

We also identified three genes (*CCL19*, *CCL21*, and *SOX9*) that might be associated with SGE in crossbreds. These genes were annotated with the response to interleukin-1 term (GO: 0070555). Interleukin-1 (IL-1) is a master regulator of inflammation by controlling innate immune processes [55]. The IL-1 superfamily includes seven pro-inflammatory proteins (IL-1α, IL-1β, IL-18, IL-33, IL-36α, IL-36β, and IL-36γ). Modulation of forebrain serotonin activity by IL-1β signaling in the dorsal raphe nucleus (DRN) was reported to control aggressive behavior, and non-aggressive mice were found to have higher levels of IL-1β in DRN than aggressive mice [56]. Therefore, we suggest that aggression behavior in pigs may be associated with SGE.

We also identified four genes (*CCL19*, *CYLD*, *MAS1*, and *NOD2*) that were annotated with GO terms associated with the regulation of NIK/NF-kappaB signaling (GO:0038061, GO:1901222, and GO:1901224) (Table 4). Diverse external stimuli, such as the release of cytokines (TNF-alpha and IL-1), viral infections, and neurotrophic factors, lead to the activation of NF-kappaB, and genes that are regulated by NF-kappaB have key roles in stress and immune responses [57]. Activation of NF-kappaB has been reported to be associated with human nervous system diseases, such as Huntington’s disease, Alzheimer’s disease, and Parkinson’s disease [58,59,60]. A prominent behavioral symptom of these neurological disorders is apathy, which is defined as the deficit of goal-directed behavior or motivational impairment [61,62]. Therefore, we suggest that these genes might be also related to SGE in pigs.

Three genes (*ADCY7*, *IL17RC*, and *NOD2*) were annotated with terms related to inflammatory response (GO:0002532, GO:0002534, and GO:1900015) (Table 4). A previous study showed that *ADCY7* was associated with depression, using both genetically modified mice and an association study of a human population [63]. *IL17RC* is essential for IL17A signaling [64], and IL17A is generally considered to cause neurodegenerative diseases by activating glial cells [65]. *NOD2* is a positive regulator of IL-1β secretion and NF-kappaB activation [66]. As noted above, previous studies have reported associations between IL-1β and aggression, and between activation of NF-kappaB and nervous system diseases; therefore, we suggest that these three genes may be related to SGE.

### 3.5. GO and KEGG Analyses for DGE

We identified the genes that were annotated with 14 and 13 significant GO terms that might be associated with the DGE in DUC and crossbred pigs, respectively (Table 5 and Table 6).

Four of the genes (*CAMK1*, *CAV3*, *FOXP1*, and *SHH*) were involved in the biological process of positive regulation of muscle cell differentiation (GO: 0051149) (Table 5 and Table 6). Growth and development of muscle are essential for the breeding of livestock species raised for meat production. Muscle formation, also called myogenesis, is a complex biological process that involves cell proliferation, differentiation, migration, myotube formation, and maturation of myofibers [67,68]. During postnatal growth, the increase in skeletal muscle mass is mainly due to an increase in muscle fiber size [69]. *CAMK1* and *FOXP1* were reported to be associated with increased lean body mass in mice [70]. *CAV3* null mice showed mild myopathic changes with the presence of necrotic fiber and variability in muscle fiber size [71]. Copy number variations in *SHH* were shown to have significant associations with body size traits in Chinese beef cattle breeds [72]. Because the growth performance of pigs is closely related to the proliferation and differentiation of muscle cells, we suggest that the positive regulation of muscle cell differentiation may be associated with the DGE for ADG in DUC and crossbred pigs. The positive regulation of muscle cell differentiation term was also identified for the SGE in DUC (Table 3). We suggest that this common GO term might be caused due to high genetic correlation (0.77 ± 0.12) between the SGE in DUC and DGE in crossbred pigs (Table 2) or the pleiotropy phenomenon, indicating that a single gene affects two or more phenotypic traits [73].

We also identified three genes (*FGF3*, *FGF4*, and *FGF19*) that were annotated with terms related to the response to the fibroblast growth factor (GO: 0071774 and GO: 0044344) and receptor signaling pathway of the fibroblast growth factor (GO: 0008543) (Table 6). Fibroblast growth factors (FGF) belong to a large protein group that is related to proliferation, migration, differentiation, and apoptosis [74]. In zebrafish, early specification of the skull was found to be regulated by *FGF3,* together with *SHH* [75]. *FGF4* has been reported to be associated with axial elongation and development of mouse embryos [76] and with Wnt signaling in mice [77]. Benoit et al. [78] reported several functions of *FGF19*, including the regulation of skeletal muscle mass through the expansion of muscle fiber, and protection of muscle from atrophy. In mice, treatment with *FGF19* caused skeletal muscle hypertrophy, and *FGF19* increased the size of human myotubes in vitro [78].

## 4. Conclusions

In this study, we conducted the estimation of genetic parameters and GWAS for the DGE and SGE on the ADG trait in DUC and crossbred pigs. Our results showed that not only DGE, but also SGE contributed to the total heritable variance in ADG. The genetic correlation between the DGE and SGE was neutral to weak in DUC and crossbred pigs, respectively. We also identified genetic correlations among the DGE and SGE on ADG for DUC and crossbred pigs, showing that the SGE of DUC was highly correlated with the DGE of crossbred pigs. The QTLs for both the DGE and SGE overlapped with previously reported QTLs associated with production- and meat-related traits. QTLs for the SGE also overlapped with QTLs associated with coping behavior and exploration during stress. Furthermore, the candidate genes (DBX1, PAX7, SHH, CCL19, CCL21, SOX9, CYLD, MAS1, NOD2, ADCY7, and IL17RC) for the SGE on ADG are associated with aggression and neurodegenerative diseases. These findings provide genomic information that will contribute to a better understanding of the DGE and SGE on ADG in pigs.

## Figures and Tables

**Table 1:** Estimates of variance components (posterior standard deviations, PSD) in purebred Korean Duroc (DUC) and crossbred pigs.

Population	Variance Components ^1^ (PSD)						
	$\sigma_{aD}^{2}$	$\sigma_{aS}^{2}$	$\sigma_{p}^{2}$	$\sigma_{TBV}^{2}$	$h^{2}$	$T^{2}$	$r_{D-S}$
DUC	1377 (352)	75 (28)	8792 (251)	1709 (378)	0.16 (0.04)	0.19 (0.04)	0.03 (0.20)
Crossbred	3410 (621)	72 (40)	9376 (433)	3625 (988)	0.36 (0.05)	0.39 (0.08)	−0.15 (0.27)

^1^ $\sigma_{aD}^{2}$, direct genetic variance; $\sigma_{aS}^{2}$, social genetic variance; $\sigma_{p}^{2}$, phenotypic variance; $\sigma_{TBV}^{2}$, total heritable variance; $h^{2}$, direct heritability; $T^{2}$, total heritability; $r_{D-S}$, correlation between DGE and SGE.

**Table 2:** Genetic correlations ^1^ (posterior standard deviations) between DUC and crossbred pigs.

Breed	Genetic Effect	DUC	
		DGE ^2^	SGE ^3^
Crossbred	DGE	0.48 (0.19)	0.77 (0.12)
	SGE	−0.53 (0.23)	−0.27 (0.25)

^1^ Genetic correlation with posterior standard deviations (in brackets) between DGE and SGE for DUC and crossbred. ^2^ DGE, direct genetic effect. ^3^ SGE, social genetic effect.

**Table 3:** Results of GO and KEGG pathway analyses associated with social genetic effect (SGE) in DUC pigs.

Term	Adjusted *p*-Value ^1^	Candidate Gene
**Inner ear morphogenesis (GO:0042472)**	**<0.05**	**INSIG1, SLC9A3R1, SOX9, USH1G**
**Regulation of protein acetylation (GO:1901983)**	**<0.05**	**CAMK1, PAXIP1, SETD5**
Regulation of systemic arterial blood pressure (GO:0003073)	<0.05	NAV2, OXTR, SOD2
Response to gamma radiation (GO:0010332)	<0.05	FANCD2, GTF2H5, SOD2
**Cytoplasmic microtubule organization (GO:0031122)**	**<0.05**	**CAV3, EZR, KIF19**
Cell differentiation in spinal cord (GO:0021515)	<0.05	DBX1, PAX7, SHH
**Neuron fate commitment (GO:0048663)**	**<0.05**	**DBX1, PAX7, SHH**
**Regulation of mesenchymal cell proliferation (GO:0010464)**	**<0.05**	**SHH, SOX9, TGFBR2**
Regulation of morphogenesis of a branching structure (GO:0060688)	<0.05	CAV3, SHH, SOX9
Positive regulation of mesenchymal cell proliferation (GO:0002053)	<0.01	SHH, SOX9, TGFBR2
Pancreas development (GO:0031016)	<0.05	SHH, SOX9, VHL
Positive regulation of muscle cell differentiation (GO:0051149)	<0.05	CAMK1, CAV3, SHH
Trachea development (GO:0060438)	<0.01	SHH, SOX9, TGFBR2
Gland morphogenesis (GO:0022612)	<0.01	CAV3, SHH, SLC9A3R1, SOX9, TGFBR2
Lung morphogenesis (GO:0060425)	<0.05	SHH, SOX9, TGFBR2
Vasculogenesis (GO:0001570)	<0.05	PAXIP1, SHH, TGFBR2
Neural crest cell development (GO:0014032)	<0.05	ERBB4, SHH, SOX9

^1^ Corrected *p*-value using Bonferroni step-down method. The most significant term per subgroup is shown in bold.

**Table 4:** Results of GO and KEGG pathway analyses associated with SGE in crossbred pigs.

Term	Adjusted *p*-Value ^1^	Candidate Gene
Regulation of protein acetylation (GO:1901983)	<0.05	BRD7, CAMK1, SETD5
**Response to gamma radiation (GO:0010332)**	**<0.05**	**FANCD2, GTF2H5, SOD2**
**Response to interleukin-1 (GO:0070555)**	**<0.01**	**CCL19, CCL21, SOX9**
Granulocyte migration (GO:0097530)	<0.05	CCL19, CCL21, IL17RC, JAGN1
Cellular response to interleukin-1 (GO:0071347)	<0.05	CCL19, CCL21, SOX9
Production of molecular mediator involved in inflammatory response (GO:0002532)	<0.05	ADCY7, IL17RC, NOD2
Cytokine production involved in inflammatory response (GO:0002534)	<0.01	ADCY7, IL17RC, NOD2
**Regulation of cytokine production involved in inflammatory response (GO:1900015)**	**<0.01**	**ADCY7, IL17RC, NOD2**
NIK/NF-kappaB signaling (GO:0038061)	<0.05	CCL19, CYLD, MAS1, NOD2
**Regulation of NIK/NF-kappaB signaling (GO:1901222)**	**<0.05**	**CCL19, CYLD, MAS1, NOD2**
Positive regulation of NIK/NF-kappaB signaling (GO:1901224)	<0.05	CCL19, MAS1, NOD2

^1^ Corrected *p*-value using Bonferroni step-down method. The most significant term per subgroup is shown in bold.

**Table 5:** Results of GO and KEGG pathway analyses associated with direct genetic effect (DGE) in DUC pigs.

Term	Adjusted *p*-Value ^1^	Candidate Gene
**Response to gamma radiation (GO:0010332)**	**<0.01**	**FANCD2, GTF2H5, SOD2**
**Positive regulation of muscle cell differentiation (GO:0051149)**	**<0.05**	**CAMK1, CAV3, FOXP1**
Negative regulation of stress-activated protein kinase signaling cascade (GO:0070303)	<0.01	AIDA, DUSP10, EZR
p38MAPK cascade (GO:0038066)	<0.01	CAV3, DUSP10, EZR
**Negative regulation of stress-activated MAPK cascade (GO:0032873)**	**<0.01**	**AIDA, DUSP10, EZR**
Negative regulation of MAP kinase activity (GO:0043407)	<0.05	AIDA, CAV3, DUSP10
Regulation of p38MAPK cascade (GO:1900744)	<0.01	CAV3, DUSP10, EZR
Lymphocyte migration (GO:0072676)	<0.01	CCL19, CCL21, MIA3
Response to interleukin-1 (GO:0070555)	<0.05	CCL19, CCL21, IRAK2
Granulocyte migration (GO:0097530)	<0.01	CCL19, CCL21, IL17RC, JAGN1
Cellular response to interleukin-1 (GO:0071347)	<0.01	CCL19, CCL21, IRAK2
Regulation of leukocyte apoptotic process (GO:2000106)	<0.05	CCL19, CCL21, TCP1, VHL
Negative regulation of leukocyte apoptotic process (GO:2000107)	<0.01	CCL19, CCL21, VHL

^1^ Corrected *p*-value using Bonferroni step-down method. The most significant term per subgroup is shown in bold.

**Table 6:** Results of GO and KEGG pathway analyses associated with DGE in crossbred pigs.

Term	Adjusted *p*-Value ^1^	Candidate Gene
Vasculogenesis (GO:0001570)	<0.01	PAXIP1, SHH, TGFBR2
**Cranial skeletal system development (GO:1904888)**	**<0.01**	**FGF4, INSIG1, TGFBR2**
**Response to gamma radiation (GO:0010332)**	**<0.01**	**FANCD2, GTF2H5, SOD2**
**Positive regulation of muscle cell differentiation (GO:0051149)**	**<0.01**	**CAMK1, CAV3, SHH**
**Mammary gland epithelium development (GO:0061180)**	**<0.01**	**CAV3, CCND1, ERBB4**
**Regulation of protein acetylation (GO:1901983)**	**<0.01**	**CAMK1, PAXIP1, SETD5**
**Neural crest cell development (GO:0014032)**	**<0.01**	**ERBB4, FGF19, SHH**
Neural crest cell migration (GO:0001755)	<0.01	ERBB4, FGF19, SHH
Melanoma (KEGG:05218)	<0.01	CCND1, FGF19, FGF3, FGF4
Gastric cancer (KEGG:05226)	<0.01	CCND1, FGF19, FGF3, FGF4, SHH, TGFBR2
Response to fibroblast growth factor (GO:0071774)	<0.01	FGF19, FGF3, FGF4
Cellular response to fibroblast growth factor stimulus (GO:0044344)	<0.01	FGF19, FGF3, FGF4
Fibroblast growth factor receptor signaling pathway (GO:0008543)	<0.01	FGF19, FGF3, FGF4

^1^ Corrected *p*-value using Bonferroni step-down method. The most significant term per subgroup is shown in bold.

## Data Availability

The dataset used in this study are available upon reasonable request from the corresponding author.

## Supplementary Materials

The following supporting information can be downloaded at: https://www.mdpi.com/article/10.3390/ani12172300/s1, Table S1: Summary of average daily gain trait in DUC and crossbreds, Table S2: QTLs identified in DUC and crossbred populations.
